# Bottom-up on-crystal in-chip formation of a conducting salt and a view of its restructuring: from organic insulator to conducting “switch” through microfluidic manipulation†
†Electronic supplementary information (ESI) available: Additional AFM images and *I*/*V* curves from the conducting AFM, SEM and EDX measurements. See DOI: 10.1039/c5sc00203f



**DOI:** 10.1039/c5sc00203f

**Published:** 2015-04-14

**Authors:** Josep Puigmartí-Luis, Markos Paradinas, Elena Bailo, Romen Rodriguez-Trujillo, Raphael Pfattner, Carmen Ocal, David B. Amabilino

**Affiliations:** a EMPA , Laboratory for Protection and Physiology , Lerchenfeldstrasse 5 , CH-9014 St. Gallen , Switzerland . Email: Josep.Puigmarti@empa.ch; b Institut de Ciència de Materials de Barcelona (ICMAB-CSIC) , Campus Universitari de Bellaterra , 08193 Cerdanyola del Vallès , Catalonia , Spain . Email: cocal@icmab.es; c WITec Gmbh , Lise-Meitner-Str. 6 , DE-89081 , Ulm , Germany

## Abstract

The chemical modification of an immobilized single crystal in a fluid cell is reported, whereby a material with switching functions is generated *in situ* by generating a chemical reagent in the flow. Crystals of the insulating organic crystal of TCNQ (tetracyanoquinodimethane) were grown in a microfluidic channel and were trapped using a pneumatic valve, a nascent technique for materials manipulation. They were subsequently reduced using solution-deposited silver to provide a conducting material *in situ* by a heterogeneous reaction. Removal of the new material from the chip proved it to be the silver salt of reduced TCNQ. Uniquely, conducting atomic force microscope (CAFM) studies show three regions in the solid. The localized original neutral organic material crystal is shown to be an insulator but to produce areas with Ohmic conducting characteristics after reduction. This inhomogeneous doping provides an opportunity for probing electrical materials properties side by side. Measurements with the CAFM witness this conducting material where the TCNQ is fully transformed to the silver salt. Additionally, an intermediate phase is observed that exhibits bipolar resistive switching typical of programmable resistive memories. Raman microscopy proves the conversion of the material in specific regions and clearly defines the intermediate phase region that could be responsible for the switching effect in related materials. This kind of “on crystal chemistry” exploiting immobilization and masking by a pneumatic clamp in a microfluidic channel shows how material can be selectively converted to give different functionalities in the same material piece, even though it is not a single crystal to single crystal conversion, and beckons exploitation for the preparation of systems relevant for molecular electronics as well as other areas where chemical manipulation of single crystals could be beneficial.

## Abstract 2

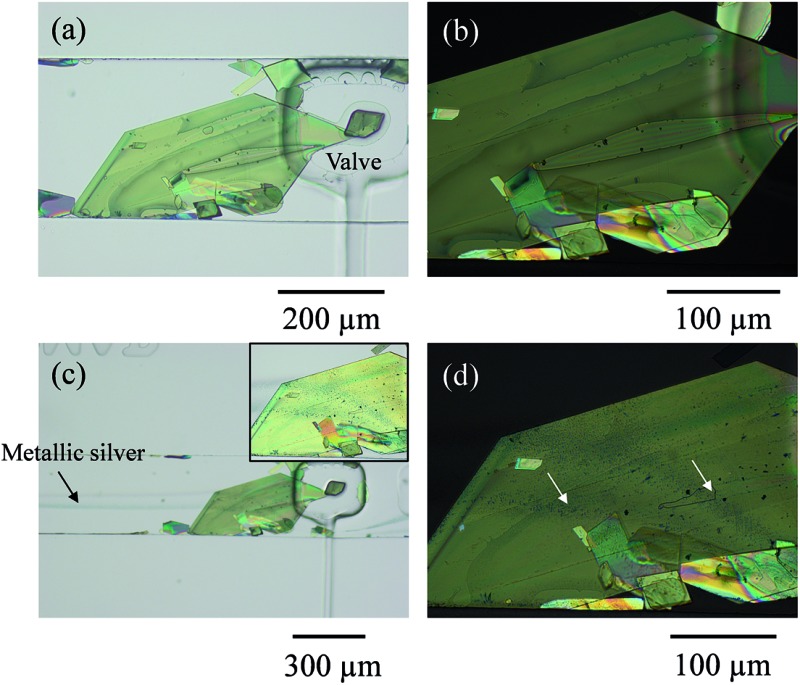
The chemical modification of an immobilized single crystal in a fluid cell is reported, whereby a material with switching functions is generated with reagent in the stream.

## Introduction

Molecular materials are presently of considerable interest particularly because of their potential use in electronics.^1^ The preparation of the systems that are capable of intrinsic electronic transport through charge carriers is frequently done by the growth of charge transfer complexes and salts, but can also be carried out by direct chemical redox reactions on the pre-formed donor or acceptor to provide a doped material, often in a mixed valence state.^2^ This approach provides a facile route to interesting materials, although the process of generating these systems and the *in situ* observation of the changes taking place in the material is in its infancy. One example is the use of vapors of oxidant to pattern conducting material.^3^ In general, though, the local generation of conducting material is challenging, and the use of relatively large crystals is difficult to carry out. It is in these areas that we wish to contribute with site specific methods for the preparation of interesting molecular materials.

The use of microfluidic chips to perform both material growth and chemical modification is a nascent but very attractive way to study the transformations of a variety of systems.^4^ Silver metal can act as a template^5^ for the growth of its salt with tetracyanoquinodimethane (TCNQ) and the conversion of a metal coordination network followed by reduction and deposition of TCNQ produced nanowires of the silver salt of this compound.^6^ In that system, pioneering in its use of pneumatic valves, the properties of the material could be studied through the use of deposition onto patterned electrodes. The resulting bundles of nanowires showed a memory effect upon addressing at different voltage sequences. That behavior is similar to the properties of the silver-TCNQ salt prepared in different ways.^7–11^

Here we show the possibility of constructing “molecular circuits” where the doped material actually acts as a macroscopic conducting component (in contrast to the previous approaches where small molecular components were obtained on electronic devices^6,12–15^) and open new vistas for molecular materials for electronics where the bulk materials form part of the devices. We use a bulk crystal as an object where conducting regions can be generated *in situ* thanks to a pneumatic clamp holding the crystals in place in the microfluidic system that also allows positioning of reagent flows. We will show how single crystals of the functional undoped precursor TCNQ can be grown and reduced in a microfluidic chip to produce a conducting material. An additional advantage of the strategy is that interruption of the process at the clamped part of the crystal would result in an integrated metal-insulating contact within a unique building block, overcoming the difficulty of controlling the electrical contacts needed for any device application. Indeed, the built-in doped–undoped interface is exploited for *in situ* electrical characterization that permits identification of an intermediate state (that we shall refer to as the intermediate phase hereafter) in the formation of the silver salt of the electron acceptor that displays resistive switching when probed by conducting atomic force microscope (CAFM).

**Scheme 1:**
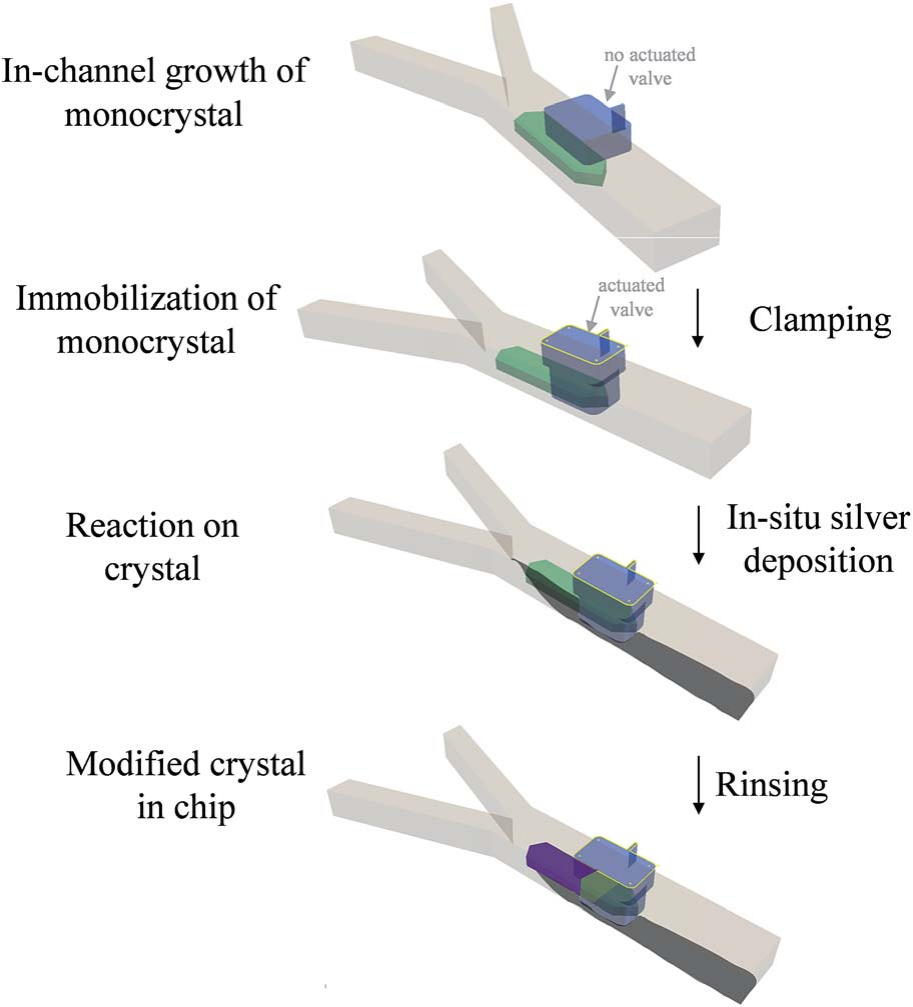
The route used for the modification of crystals in a fluid channel with pneumatic clamps for immobilizing and masking and area of it. The green object represents the original crystal grown in the chip, the purple area the modified material and the light green area an intermediate zone where partial reaction could take place.

The general approach is shown in Scheme 1. It consists of growing a crystal in the microfluidic channel, immobilizing it with a pneumatic clamp and subsequently performing chemistry on the solid with solution-born reagents (see ESI† for further details). This approach is more controlled than a bulk method because microscopic observation of the doping process *in situ* is allowed (difficult with a dipping process), clamping of the crystal allows precise positioning of solvent flow with respect to the object, very precise mass transport conditions and concentration of reagents are achieved because of the laminar flow in the channel that avoids capillary effects on the trapped object, very small amounts of material are used making the process less wasteful than bulk methods, and cleaning *in situ* can be performed thereby avoiding manipulation that might lead to contamination. Though the material under the activated clamp is protected from the reagents because it is a physical barrier, we shall see that diffusion of Ag through the silver-TCNQ^7^ is still possible to some extent (intermediate region) to the bulk lattice of the clamped TCNQ. This fact also allows monitoring of the induced changes at the interfaces between areas depicted in the scheme. The areas where the treatment is carried out can be varied according to the position of the flow in this channel, as shall become clear in the Results and discussion section that follows.

## Results and discussion

The microfluidic chips used for these experiments comprise two layers, one where the fluids combine under laminar flow conditions and another having voids where gas can be pressurized to deform the intermediate poly(dimethylsiloxane) (PDMS) membrane that acts as a clamp.^16,17^ We used the same approach to trap silver deposited in the channel as a coordination polymer.^6^ Here we perform the reverse process: we grow a macroscopic crystal of an organic compound – TCNQ – then immobilize it with the clamp (in the process covering part of it) and then let react the crystal with a reagent – silver metal – generated in the channel at stream boundaries. Release of the clamp leaves the crystal free in the channel. The transparent chip allows *in situ* monitoring of crystal growth and modification in real time and space using an inverted optical microscope with polarising filters incorporated.

**Fig. 1:**
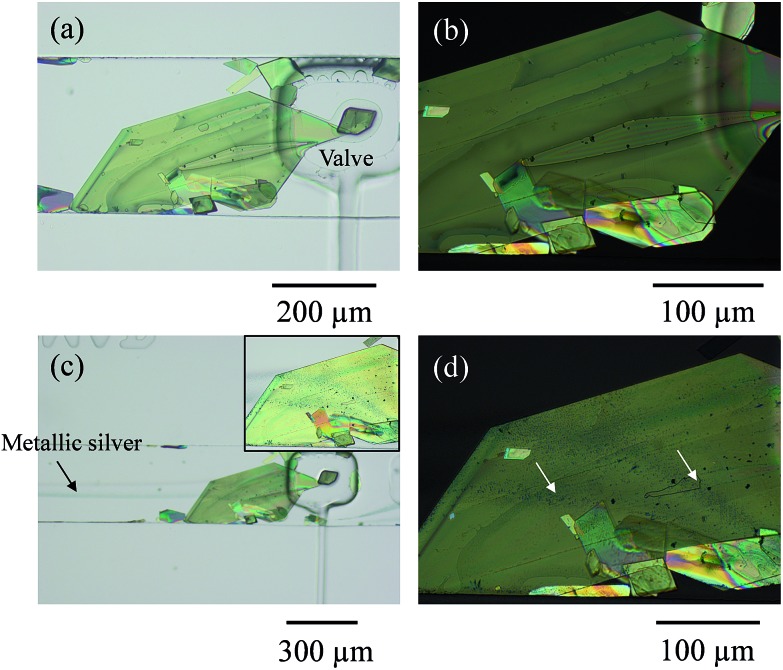
(a) Optical micrograph of a large TCNQ crystal (and other smaller ones) grown in a microfluidic channel and trapped underneath a pneumatic valve to avoid its movement during the flow of reagents, and in (b) an enlarged polarized optical micrograph of the same object. In (c), the valve is actuated, and a flow of aqueous silver(i) and aqueous reducing solution generating silver metal can be appreciated as a shadowy line coming from the left-hand side of the image. The inset image in (c) shows the two reductive pathways generated onto the TCNQ crystal which are indicated with arrows in the polarized micrograph (d) where the single crystal nature is clearly maintained.

Two inlet channels were used, as in our previous work.^6^ On this occasion a saturated solution of TCNQ in acetonitrile was introduced into one side of the main channel and water was injected under controlled conditions through the other inlet with the help of a syringe pump system. When the water stream contacted the TCNQ solution, TCNQ crystals could be generated at the interface of the two co-flowing streams *via* anti-solvent precipitation. Controlling the flow rate of both, the TCNQ solution in acetonitrile and the water stream, TCNQ crystal size could be adjusted by solvent/non-solvent precipitation (see ESI† for further details and Fig. SI1†). Fig. 1 shows the largest crystal that was grown under these conditions, and the one that was used for the subsequent experiments. The crystal was immobilized by actuating the rectangular valve clamp that can be seen on the right hand side of the crystal in the micrographs in Fig. 1.

In order to dope the TCNQ to generate the conducting Ag-TCNQ salt, an aqueous solution of silver(i) salt was flowed through one channel and an aqueous reducing solution was flowed through the other. Metallic silver is generated at the interface of these solutions (as seen in Fig. 1c) and comes into contact with the TCNQ crystal and reacts heterogeneously generating the salt of the organic electron acceptor spontaneously.

It is clear that the presence of both the crystal and the actuated valve change the course of the fluid flow, but the lamellar flow conditions are largely maintained. Enough silver was generated under these conditions that the whole of the crystal exposed to the fluid could be doped to form Ag-TCNQ. Initially, this doping was observed optically in the microscope.

**Fig. 2:**
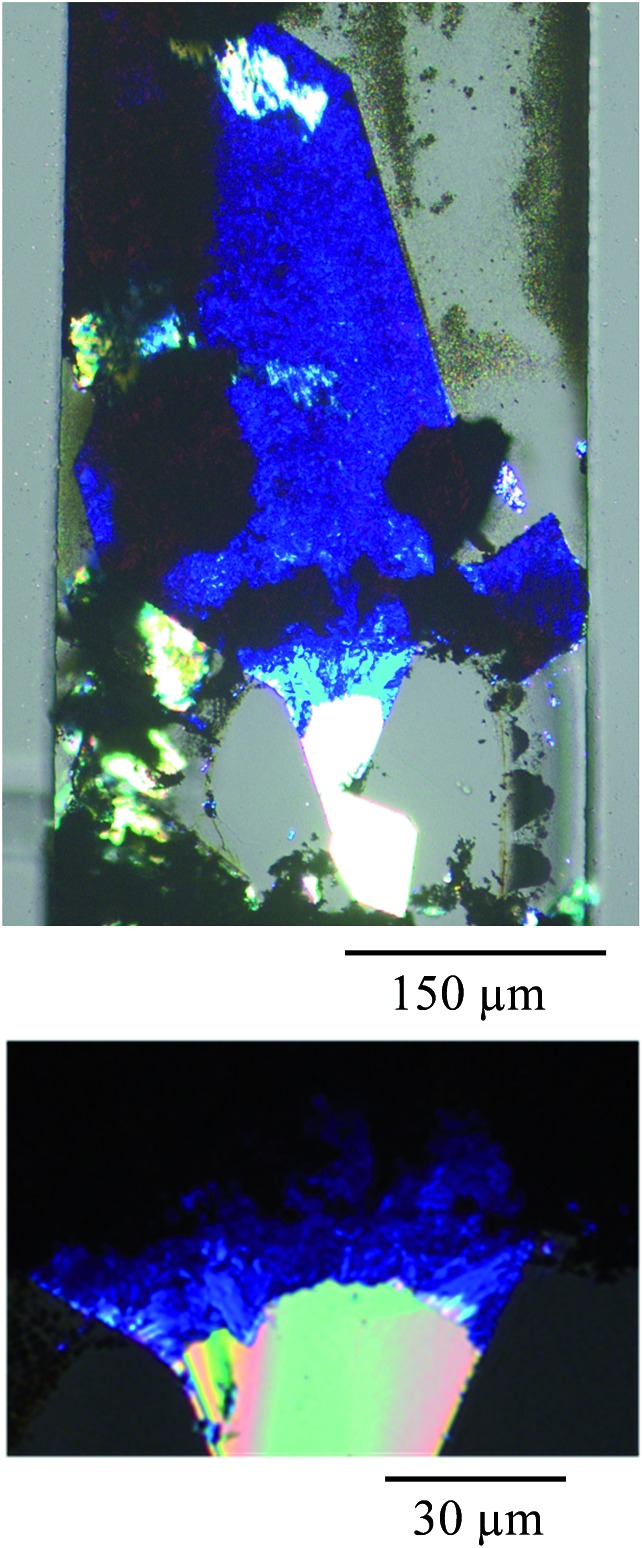
Polarized optical micrographs of the Ag(i) doped TCNQ crystal (from that shown in Fig. 1). The top image shows the complete object and the lower one the area between doped and undoped regions showing the crystalline and polycrystalline regions. The white areas correspond to an unreacted region of the crystal of TCNQ, while light and dark blue regions correspond to the formation of the Ag-TCNQ complex. The black regions correspond to metallic silver.

Fig. 2 shows a micrograph obtained with polarizer and analyzer crossed where the undoped part of the TCNQ crystal under the clamp appears bright white because of the high contrast in the image. In fact, starting from the outermost crystal end (top end in the figure) to the clamped end, different regions were observed whose structures are clearly distinct from one another. The Ag-TCNQ is seen as dark blue material, and in between these two regions there is an intermediate lighter blue that is also a silver-containing complex. This intermediate phase material is also birefringent, indicating a degree of structural order. Analysis of the material using scanning electron microscopy (SEM) and energy-dispersive X-ray spectroscopy (EDX) confirmed the location of the silver in the intermediate region, and the absence of silver in the clamped region of the TCNQ crystal (see ESI Fig. SI6–SI8†). The undoped TCNQ region shows no evidence for the presence of silver, while the intermediate region does show peaks corresponding to the element in the EDX spectra. It is clear from the polarization of the light that an ordered material is obtained, although this is clearly not a single crystal to single crystal transformation: in the close-up optical image of the material after doping in Fig. 2 the object is evidently polycrystalline.

The nature of this composite material object that results from doping the TCNQ crystal was first probed by AFM as well as conducting atomic force microscopy (CAFM), after removal of the crystal from the chip and over the different regions. We shall see that the inhomogeneous doping actually provides an opportunity to probe the electrical properties of the different materials produced side by side under the CAFM, without the need to remove samples and compare different preparations with the consequent doubt of reproducibility of conditions. The crystal was contacted by attaching a copper wire to the crystal using graphite paste at the conducting end, which acts as fixed electrode while the metallic AFM tip can be viewed as a mobile electrode to which the voltage is applied. Prior to the conducting measurements, the appropriate conditions of applied voltages were checked to avoid structural damage as the observed in other organic materials under applied electric fields.^18^ The residual material from the microfluidic process that was weakly attached to the material's surface was evident. To obtain noisy free imaging, this debris was removed by gentle tip scanning to uncover the different regions without any visible damage on their surface structure. Scanning the AFM tip at low controlled load to avoid noisy images while verifying no structural changes in the material during the process was performed in every area (ESI, Fig. SI2†).

**Fig. 3:**
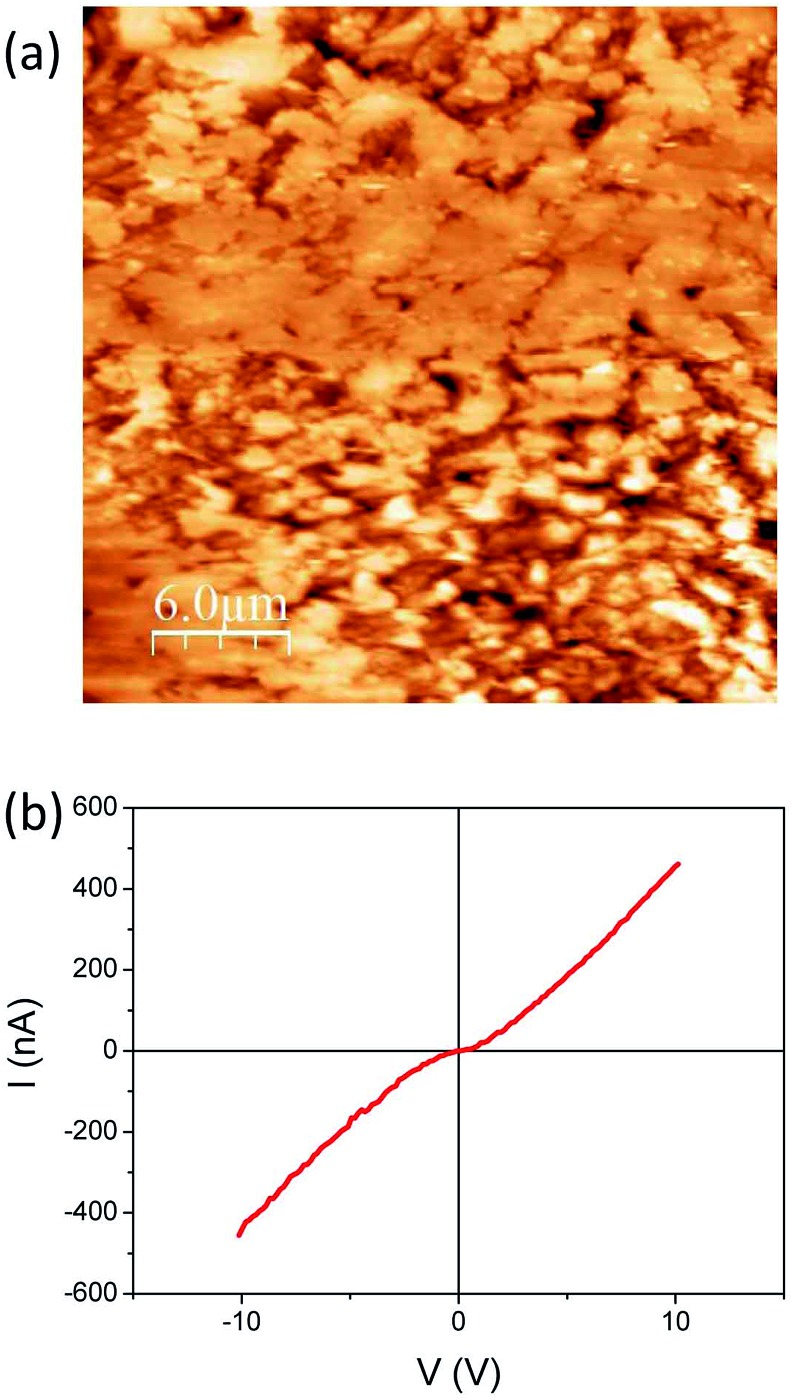
(a) Topographic AFM image obtained in contact mode on the conducting region of the sample and (b) a typical *I*/*V* sweep at a given point on this region.

**Fig. 4:**
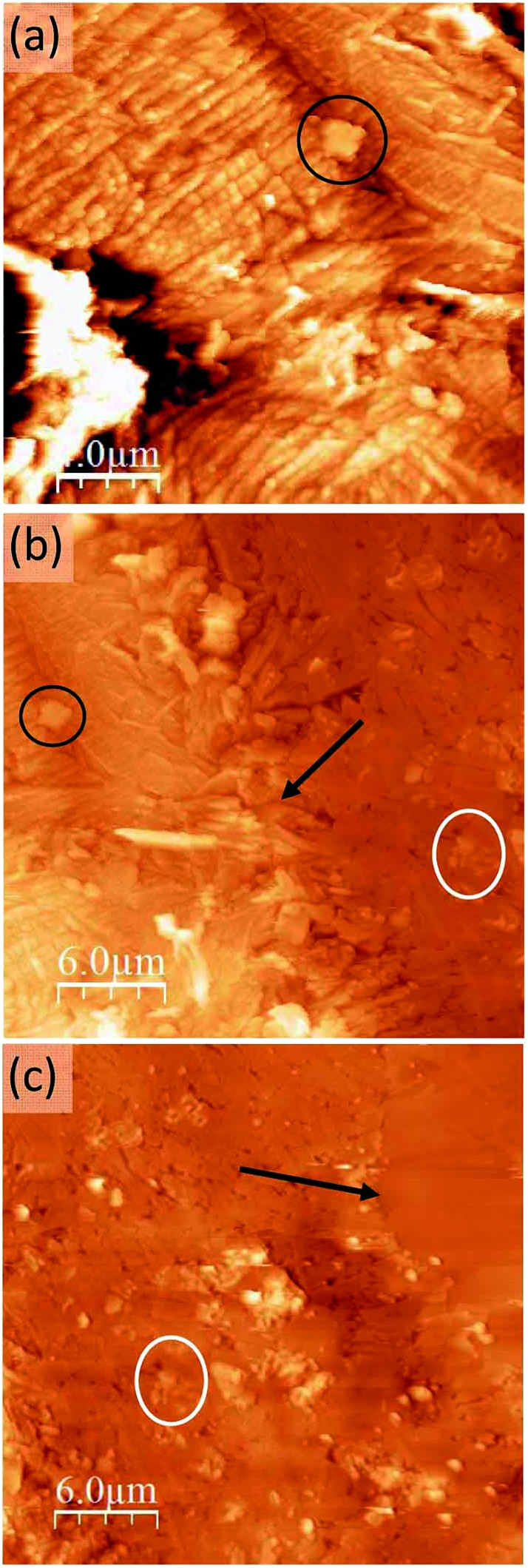
Topographic AFM images at different locations of the sample: the growth front of the conducting region (a) at the boundary between the conducting region and the intermediate region (b) and at the boundary between this intermediate region and the pure TCNQ part of the crystal which can be identified as the flatter region at the upper corner (c). Here the orientation of the crystal is rotated 90° anticlockwise with respect to Fig. 2. Some common features and the two inter-region boundaries have been marked (circles and arrows, respectively) as surface location references.

Focusing on the conducting (dark blue in Fig. 2) region where Ag-TCNQ is formed, the topographic image shows a relatively rough surface with a granular appearance (Fig. 3a) and an rms roughness of about 80 nm. In general the grains or plates that appear are smaller than 5 microns. The conductivity map (ESI, Fig. SI3†) demonstrates that the current passes through these crystallites that must be interconnected throughout the material for bulk current to pass. The *I*/*V* response in this material (Fig. 3b) shows a nearly linear curve with a subtle inflection around zero voltage, indicative of a slight gap, probably caused by the polycrystalline nature of the obelisk in which inter-grain contacts are prevalent. Nonetheless, the conductivity is remarkable given that this block of material is generated from a single crystal that is totally restructured upon its reaction with silver. Close to the intermediate region, the growing front has a highly anisotropic morphology (Fig. 4a) that reminds us of the uniaxial growth observed for Ag-TCNQ nanowires^7^ in which silver apparently diffuses through the Ag-TCNQ or undergoes Ag exchange with that in the Ag-TCNQ complex along the growth direction to react with the pure TCNQ.

**Fig. 5:**
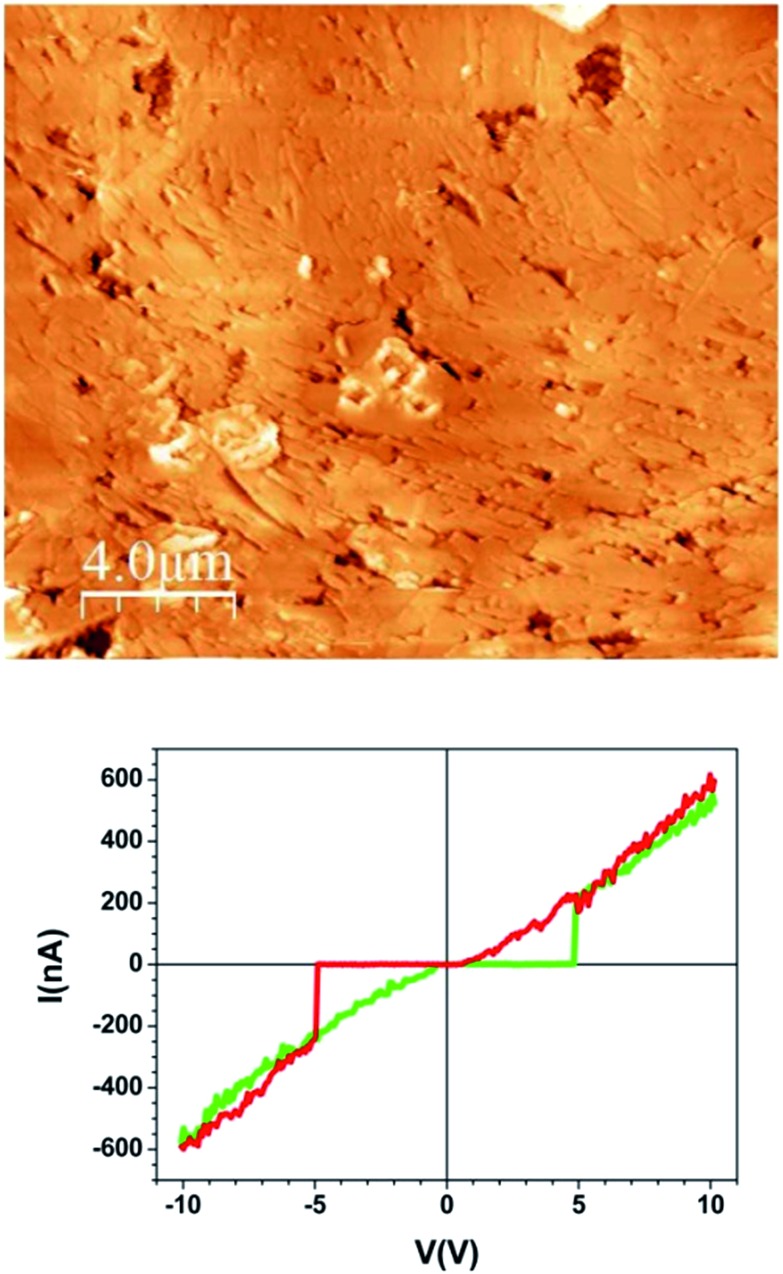
Topographic AFM image of the intermediate material region of the sample and a typical *I*/*V* sweep over this region.

Between this more conducting region and the insulating unmodified TCNQ region the material shows a very different texture (Fig. 4b right and 4c left). The topographic AFM images reveal flat elongated crystallites on the surface that are oriented apparently at random with respect to the original crystal orientation (Fig. 5, top). The roughness (35–40 nm including inter-grain holes) is significantly lower than in areas where total conversion to the conducting phase has taken place. As revealed by the current characteristic curves measured by CAFM at specific points, the intermediate phase material in this area displays a bipolar resistive switching (RS).

Several factors can be observed from the representative *I*/*V* curve (Fig. 5, bottom). First, as the voltage sweeps from zero to positive values the current does not flow until approximately 5 V when there is a jump to around 200 nA. The current then increases linearly with increasing potential. When the potential is reduced to zero the current again follows a linear arrangement even below 5 volts until it meets 0 nA around 0 V. This differing behaviour in forward and reverse sweeps means that a bistability is present. Moreover, the curve is nearly symmetric for positive and negative voltages showing current rectification.

The two abrupt transitions result in switching from a high-resistance state (HRS) to a low-resistance state (LRS) occurring at threshold voltages *V*_th_ (≈± 5 V). Remarkably this is not a conventional RS cycle. On the one hand, the current follows a highly linear behaviour (Ohmic) in the LRS while the HRS is highly insulating so leakage-current is suppressed. On the other hand, the *I*/*V* curve is a non-conventional or non-crossing counter-clock-wise cycle that has been observed in several systems^19^ and modelled as two back-to-back Schottky barriers connected in series. In our case, these two barriers correspond to the two interfaces between the electrodes (conducting Ag-TCNQ and metallic AFM tip) and the switching material (intermediate composite). The bipolar character of the process is demonstrated by performing successive minor loops (ESI, Fig. SI5†) which show that after an initial HRS to LRS transition (forming cycle), the system remains at LRS as the sign of the voltage is not reversed.

The switching behaviour in the silver salt of TCNQ has been witnessed both in bulk and nanostructured samples (prepared in very different ways) before.^7–11^ The phenomenon has been assigned to a reversible redox reaction induced by the local electric-field where silver metal and neutral TCNQ are formed at the interface, and mobility of silver ions has been inferred in this process^20^ and could well be happening in our experiments where the AFM tip is in contact with the material's surface.

However, none of these cases have imaged the restructuring of the material and the change of oxidation state of the components. Here, the CAFM measurements show clearly the zones where the redox change in the material is taking place, where the Ohmic conductivity is and where the material that shows resistive switching is.

**Fig. 6:**
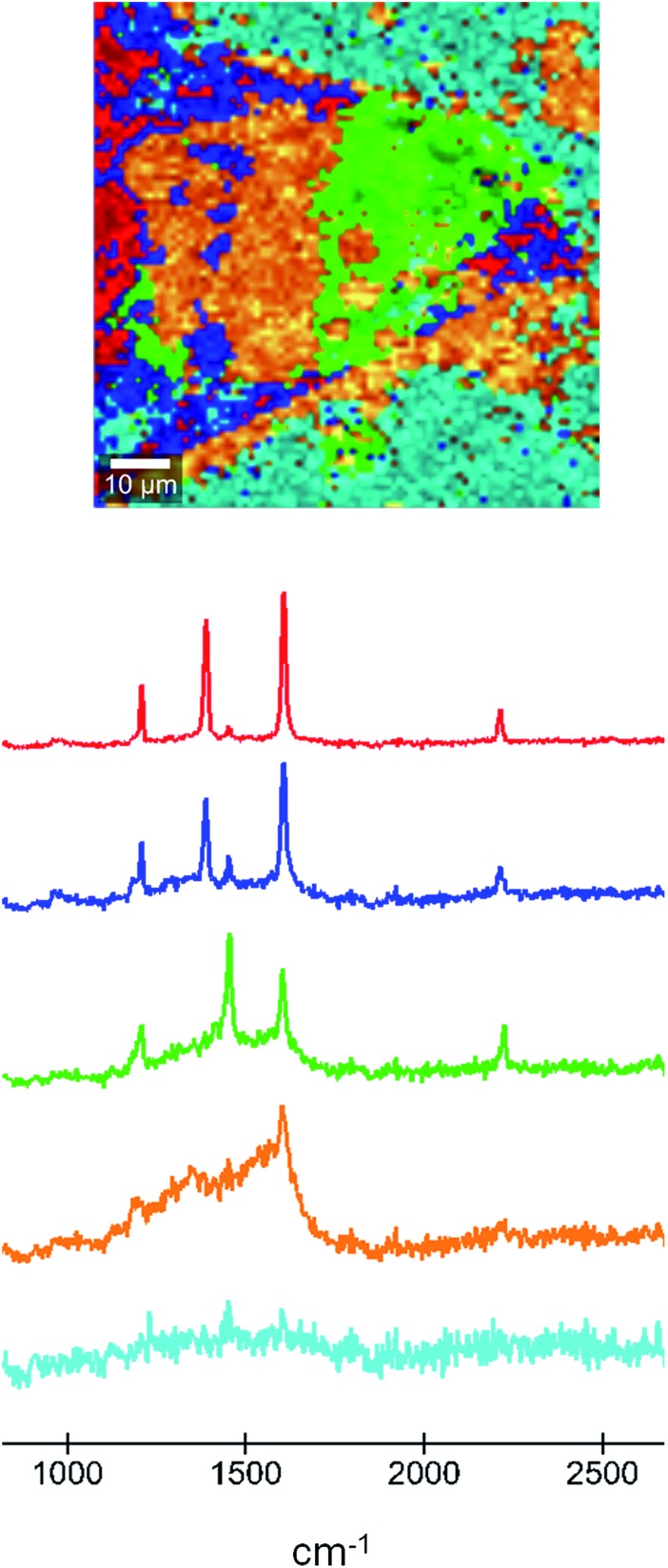
A Raman micrograph (top) of the doped TCNQ crystal with the areas corresponding to each colored Raman spectrum (below). The green area corresponds to neutral TCNQ and the red area to the full charge transfer salt. The light blue area is the background.

In order to shed light on the chemical nature of the area that shows resistive switching we performed Raman microscopy on the sample that had been used in the AFM experiments. In the Raman microscopy images we can associate this change in property with the oxidation of the TCNQ component in each area. In this technique, areas with a particular Raman spectrum can be identified, allowing the determination of their different nature. A Raman map micrograph and the corresponding spectra of the material formed in the microfluidic chip after doping of the TCNQ crystal are shown in Fig. 6. The red area and curve correspond to the fully doped region, where the Ag-TCNQ salt is formed. The Raman spectrum shows the typical peaks of this salt, at approximately 1202, 1383, 1603 and 2210 cm^–1^. The green area, with Raman peaks at 1202, 1450, 1603 and 2225 cm^–1^, corresponds to the undoped TCNQ. The bands located at 1202 cm^–1^, 1603 cm^–1^ and 2225 cm^–1^ are associated to C=CH bending, C=C ring stretching and C–N stretch modes, respectively. The most obvious and characteristic change is that of the peak at 1450 cm^–1^ in the neutral material to 1383 cm^–1^ in the silver salt. This shift in the Raman band can be attributed to variations of the C–CN wing stretch vibration mode between TCNQ and Ag-TCNQ, as previously reported by others with the synthesis of Cu-TCNQ from TCNQ.^21^ The light blue area is the background.

The orange area in the Raman micrograph corresponds to the light blue region in the optical micrographs and is that which displays resistive switching. The Raman spectrum corresponding to this region shows a remarkably broad signal in the region of around 1200 to 1650 cm^–1^, with the only clear peak being at 1603 cm^–1^. Therefore, the structure of the TCNQ in this region is not well defined and must comprise a mixture of neutral and charged molecules. We believe that this area must contain coordination oligomers of TCNQ with silver in a non-ordered state. The observation of resistive switching, as noted above, is likely to be caused by movement of silver metal atoms across the surface, and this disordered material is clearly a convenient route for this process to take place. It is interesting to see that a fourth region, presented in dark blue in Fig. 6, that shows a Raman signal that is very similar to the salt but with a more dominant contribution from an undoped TCNQ signal. This region can be present within areas of intermediate phase material, and we hypothesize that an inter-conversion could take place here that could explain the switching behaviour.

## Conclusions

The results presented prove that microfluidic chips can serve as a platform for performing local chemistry on crystals, in the present case converting an insulating material into a conductor and also a switchable material. A degree of chemical control in the doping process despite the fact that the reaction is clearly a heterogeneous one. The flow characteristics and concentrations can be tuned so as to modulate the degree of conversion, and the use of the pneumatic clamp ensures immobilization of the initial material as well as partial masking of areas of the crystal so as to have structural and electrical reference.

We have also shown that the method allows identification of an intermediate phase present in the doping process, where a birefringent material containing silver displays resistive switching can be identified. This observation is important for the kind of material presented here, as it may mean that the resistive switching in this kind of salt could arise from charge injection into/from interface states where an intermediate phase material could be important. The observed current rectification and leakage current suppression are valuable to avoid cross talk between eventual memory devices in high density applications.

## Experimental

The chemicals were purchased from Sigma-Aldrich Co. and were used without further purification. High purity solvents were purchased from Teknokroma, and were used as received. Deionized Millipore Milli-Q water was used in all experiments. The microfluidic chips used in this study were prepared as detailed elsewhere.^6^

In CAFM, the conducting tip acts as a movable electrode which is placed in direct contact with the sample under controlled load, *i.e.* by using a normal force feedback, while measuring the current between tip and sample. In our set-up, the current is measured between the biased AFM tip and a metallic counter-electrode attached to the crystal under study which is directly contacted to ground. The conducting response of the samples was obtained following two different strategies: (i) simultaneously acquiring topographic images *z*(*x*,*y*) and current maps *I*(*x*,*y*) over a given region at a given voltage, and (ii) acquiring *I*–*V* curves at selected (*x*,*y*) locations on the surface (*e.g.* different composition regions). Scanning force microscopy (SFM) measurements were performed under low humidity conditions (<5% RH, obtained by a continuous N_2_ gas flux) using a commercial head and software from Nanotec.^22^ CrPt coated Si tips from Budget sensors with nominal stiffness of 40 N m^–1^ were used.

Raman spectra and images were recorded with a WITec Confocal Raman Microscope (WITec alpha 300R, Ulm, Germany) which consists in a Confocal Microscope with a high throughput Raman Spectrometer. For the Raman image, the crystal was scanned with a piezo-electric stage, using a 60*×* water immersion microscope objective (NA = 1) and a 532 nm (frequency-doubled Nd:YAG laser) as excitation source.

The TCNQ crystal was examined within a scan range of 85 × 85 μm^2^ and 85 × 85 pixels (=7225 spectra). Integration time was 50 ms per spectrum, *i.e.* the acquisition time per image was approximately 7 minutes.

The data set was evaluated using the WITec Project software by k-means cluster analysis. For this Raman image 5 clusters were generated (as shown in Fig. 6).
