# Supplementary material for: Bottom-up on-crystal in-chip formation of a conducting salt and a view of its restructuring: from organic insulator to conducting “switch” through microfluidic manipulation
Source: Chem Sci. 2015 Apr 14;6(6):3471–7. doi: 10.1039/c5sc00203f (PMC5492874; doi:10.1039/c5sc00203f)
Supplement: Supplementary file 1 [file SC-006-C5SC00203F-s001.pdf]

Electronic Supplementary Material (ESI) for Chemical Science.
This journal is © The Royal Society of Chemistry 2015

ELECTRONIC SUPPORTING INFORMATION FOR

Bottom-up on-crystal in-chip formation of a conducting salt and a view of its restructuring: From organic insulator to conducting "switch" through microfluidic manipulation

Josep Puigmartí-Luis,[a*] Markos Paradinas,[b] Elena Bailo,[c] Romén Rodriguez-Trujillo,[b] Raphael Pfattner,[b] Carmen Ocal,[b,*] and David B. Amabilino[,b,§,*]

[a] EMPA, Laboratory for Protection and Physiology, Lerchenfeldstrasse 5, CH-9014 St. Gallen, Switzerland
[b] Institut de Ciència de Materials de Barcelona (ICMAB-CSIC), Campus Universitari de Bellaterra, 08193 Cerdanyola del Vallès, Catalonia, Spain
[c] WIiTecGmbh, Lise-Meitner-Str. 6, DE-89081, Ulm, Germany
[§] Present address: School of Chemistry, The University of Nottingham, University Park, NG7 2RD, UK.

Details of the solvent/non-solvent precipitation method employed inside the microfluidic platform:

The microfluidic platform consists of two inlet channels and an outlet flow. In a typical experiment, a saturated solution of TCNQ in acetonitrile was introduced into one inlet channel and water, a non-solvent for TCNQ, was injected through the other inlet. The whole process was carried out at room temperature (approximately 20ºC). The flow rates of both solutions were precisely controlled with the help of a syringe pump system. By controlling the flow rates of the TCNQ solution and the water flow, we could control the localization of the diffusive mixing zone between the two streams where the crystallization took place. The flow rates were varied between 1 $\mu Lmin^{-1}$ to 10 $\mu Lmin^{-1}$. We observed that increasing the flow rate, the width of the diffusive mixing zone decreased, and hence small TCNQ crystallites were generated. The clamping of TCNQ crystals was conducted by applying a pressure of 3 bars to the control layer. This pressure ensured an effective masking of the TCNQ, and hence, it allowed a favourable condition for the generation of an intermediate crystal state. After the electroless deposition of silver onto TCNQ, a subsequent washing step with deionized (DI) water was conducted to enable an efficient and straightforward removal of all surplus reagents present inside the microfluidic channel. In this case, a DI water flow was injected during 3 to 5 minutes to ensure a complete removal at a flow rate of 5 $\mu Lmin^{-1}$ approximately.

Moreover, the microfluidic channel aspect ratio was optimized to ensure both: i) a non-clogging condition for the formation of TCNQ crystals, and ii) the subsequent chemical treatment of the trapped TCNQ crystals with silver and under laminar flow regime. The silicon wafers employed in the fabrication of the microfluidic platforms and the two-layer microfluidic chip assembly were fabricated using conventional soft lithography techniques and processes described elsewhere.[1] Finally, the dimensions of the microfluidic channel are 50 μm × 300 μm × 1 cm (height × width × length).

We observed that the solvent/non-solvent precipitation of TCNQ could be better controlled in microfluidic channels that were 300 μm in width, i.e. in this case it was possible to generate

[1] B. Z. Cvetković, J. Puigmartí-Luis, D. Schaffhauser, T. Ryll, S. Schmid, P. S. Dittrich, *ACSNano*, **2013**, *7*, 183-190.

crystals that will not obstruct the laminar flow condition required for the subsequent chemical treatment. Nonetheless, we observed that employing this microchannel aspect-ratio, small crystals could form which will not facilitate the formation of an intermediate region in the TCNQ crystal; one of the aims of the present study. We observed that only AgTCNQ structures are generated when small TCNQ crystals are treated with silver (see Fig. SI1(d)).

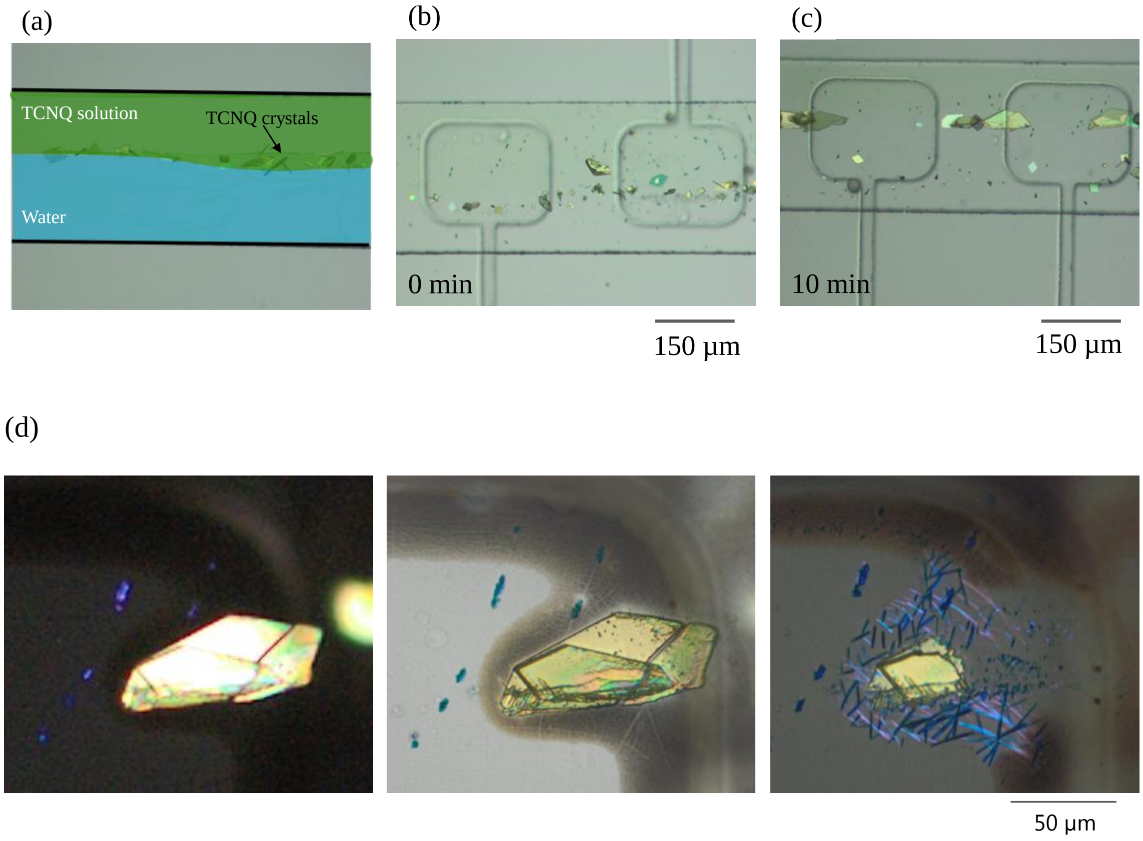

Figure SI1 (a) Schematic drawing of TCNQ crystals formation generated at the interface of the two co-flowing streams via anti-solvent precipitation. (b) and (c) micrographs showing small and bigger TCNQ crystals generated at the interface of the two reagent streams, respectively. As shown in (c), by precisely controlling the TCNQ solution and the water stream bigger crystals could be generated after 10 minutes in a typical experiment. (d) AgTCNQ wires generated from the chemical reduction of smaller crystals of TCNQ.

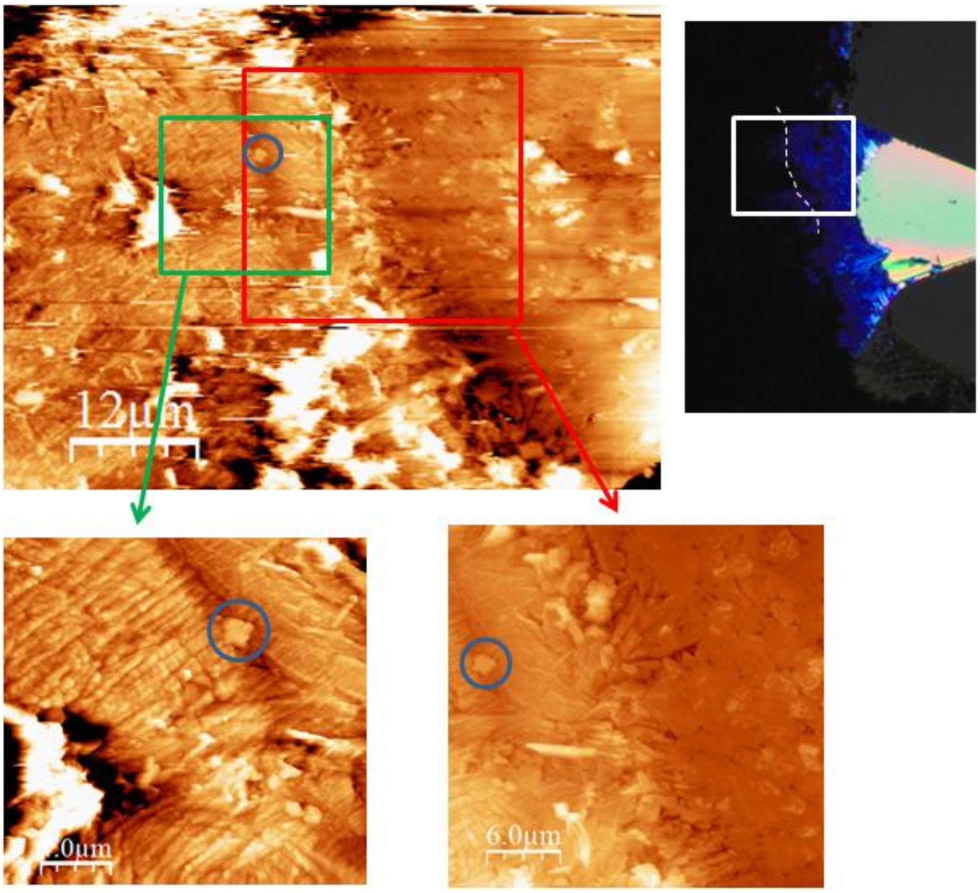

Figure SI2. Top: AFM topography (left) of the region marked in the optical micrograph (right). The dashed white line in the later has been drawn in the optical micrograph to identify the boundary between conducting AgTCNQ (dark blue) and the intermediate region (see the paper body text) which was scanned in the AFM image. The material weakly attached to the surface is rest of the reactants within the microfluidic cell. To obtain noisy free imaging, this debris is removed by gentle tip scanning to uncover the different regions without any visible damage on their surface structure. The same surface feature has been circled in all images to have a reference for surface location.

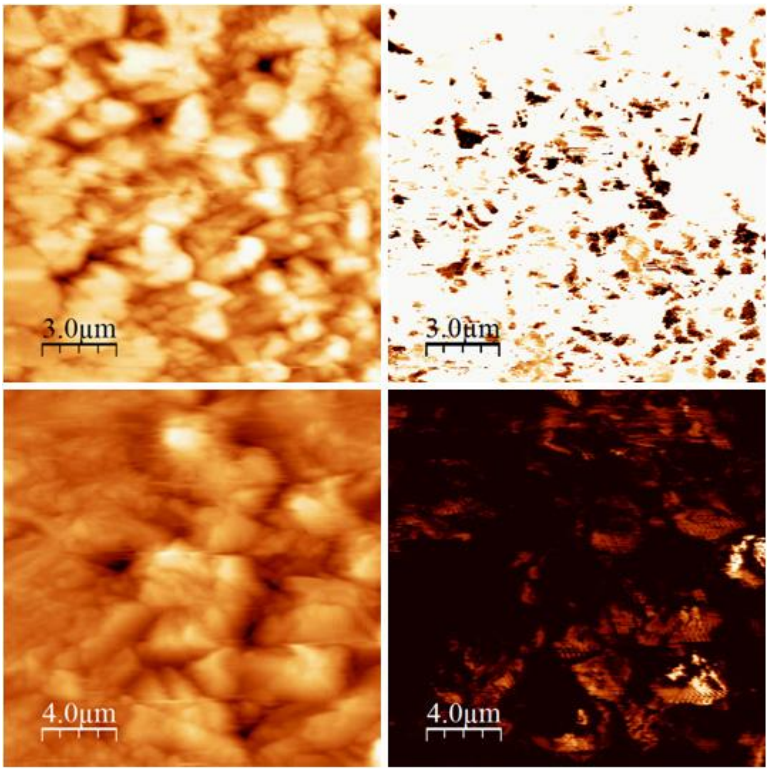

Figure SI3. Topography and simultaneous current maps obtained at V_{ip}= -10V before cleaning (top) and at V_{ip}= 0.1V after cleaning by repeated tip sweep (bottom) over the conducting region (AgTCNQ) of the modified crystal. The surface is formed by grains which provide a relatively large roughness and present current values at much lower voltages after surface cleaning and a nearly linear behavior as a function of voltage (see Figure 3b in the paper main text). Note that whereas for topographic images the color code is the commonly used, bright for high and dark for low, for the current maps it depends on the voltage sign. Thus higher currents appear darker in CSFM images taken at negatives V_{tip}, while brighter for positives V_{tip}.

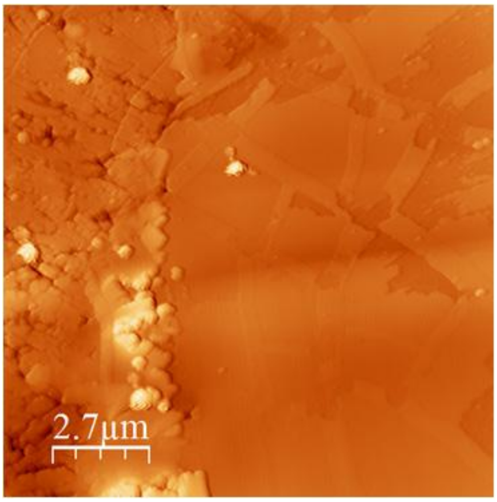

Figure SI4.AFM topography at the boundary between the intermediate region (presenting resistive switching) and the non-reacted TCNQ. Note the different structure consisting of small grains randomly distributed forming the intermediate compound and very flat crystallites of the original TCNQ crystal.

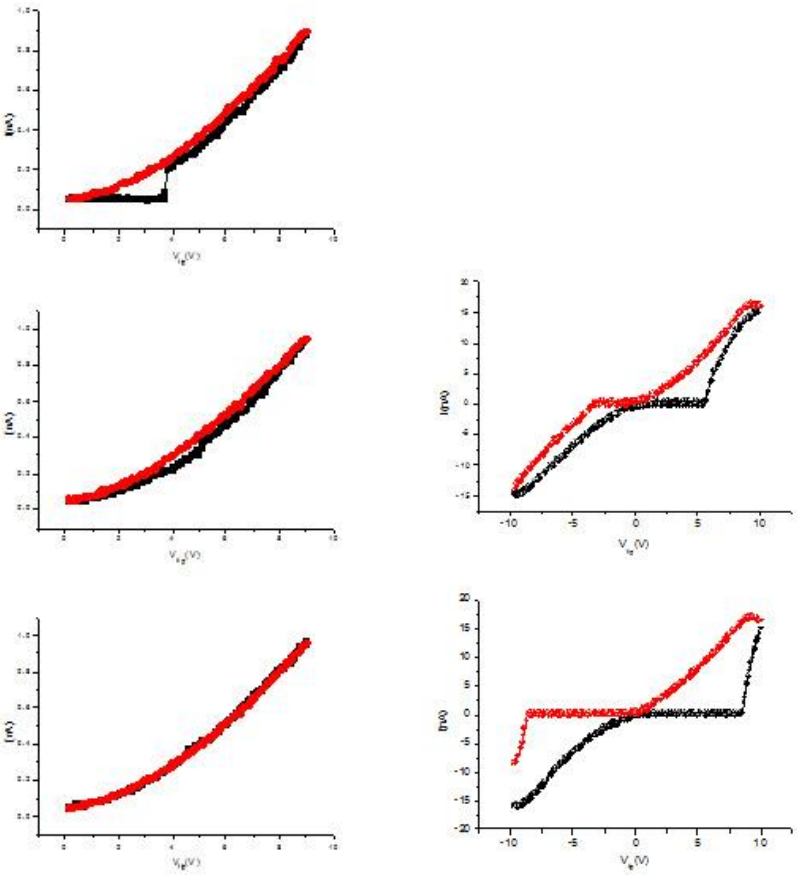

Figure SI5. Left:Minor I/V curves, i.e. measured current as a function of tip voltage (V_{tip}) only for positive values. After the first transition from high resistive state (HRS) to low resistive state (LRS) shown in the top panel, for consecutive voltage sweeps, the cycle quickly closes until the system remains in this LRS, i.e. to reverse the resistance state back lo HRS, it is necessary to change the voltage sign is needed: bipolar switch.Right: Bipolar RS loops illustrating the non-crossing and rectifying characteristics of interface states processes.

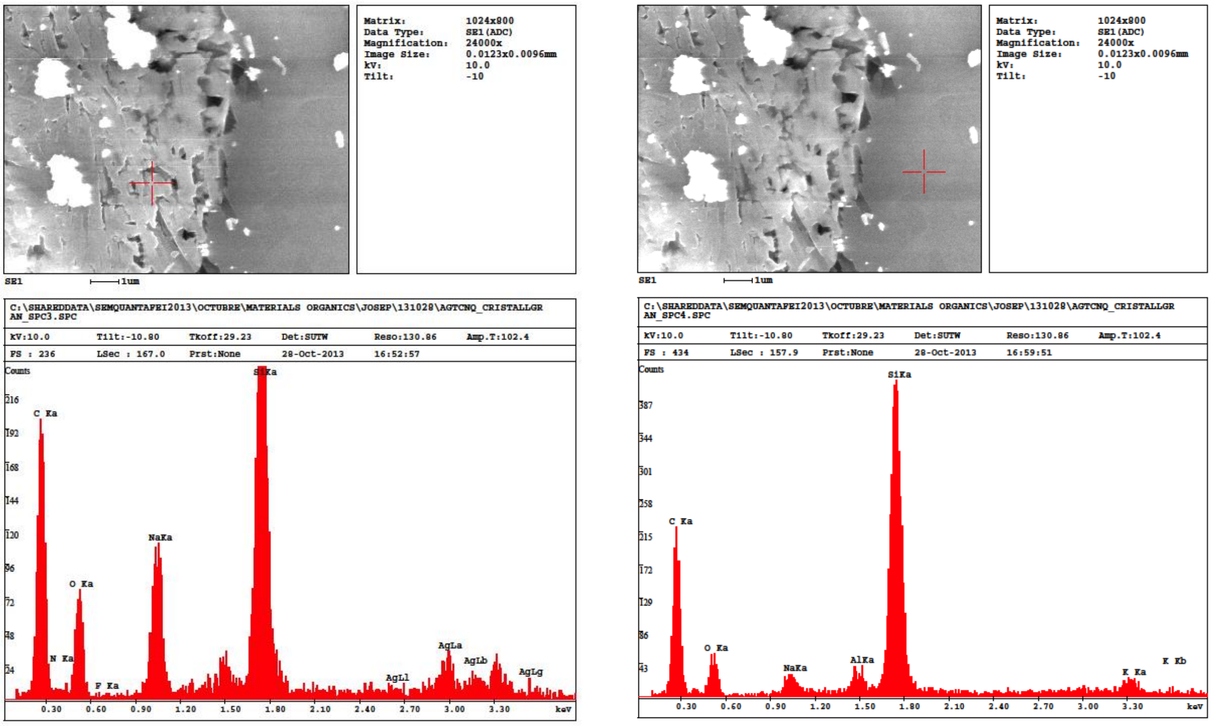

Figure SI6. SEM images of the interphase between undoped TCNQ and the intermediate doped material (top) and the EDX spectra (bottom) corresponding to the areas marked with the red cross in the images.

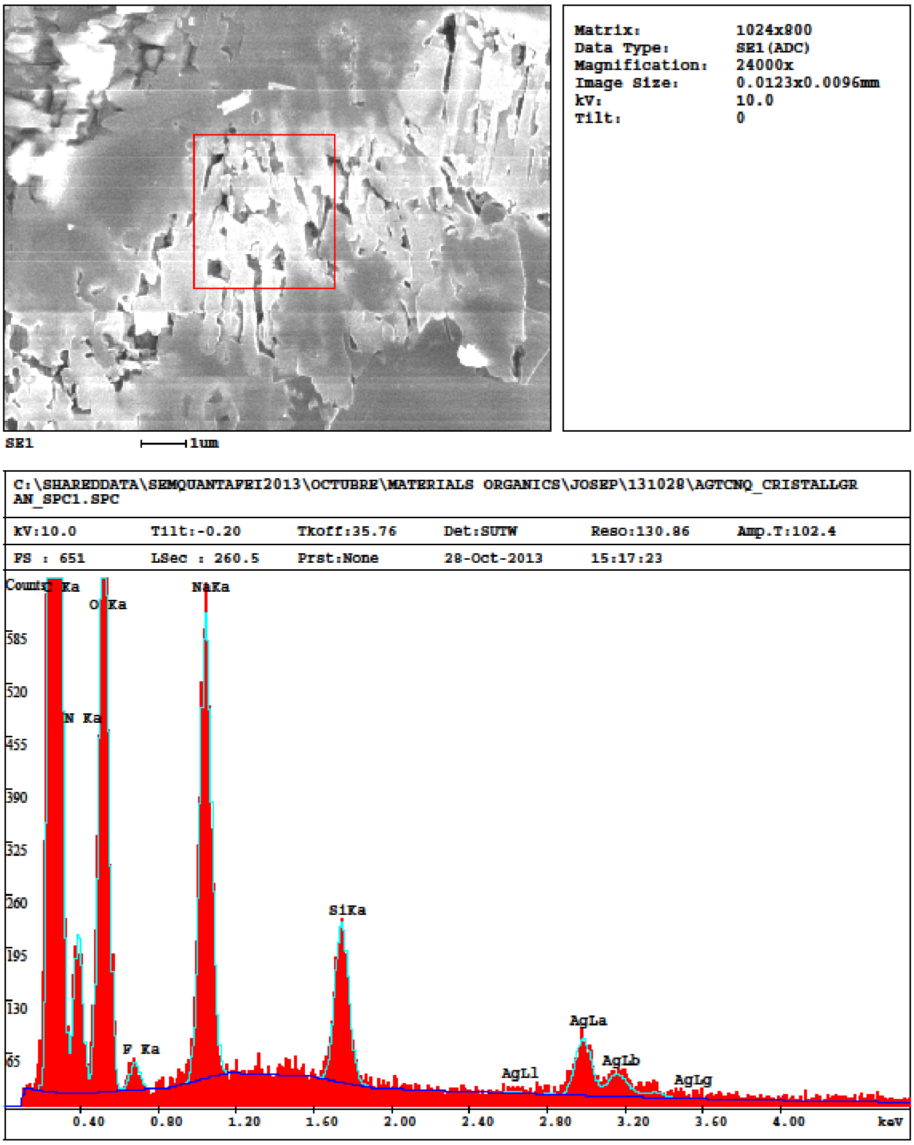

Figure SI7. In the top SEM image of the wire-like morphology area, AgTCNQ section, and EDX spectra of the region indicated in the SEM image.

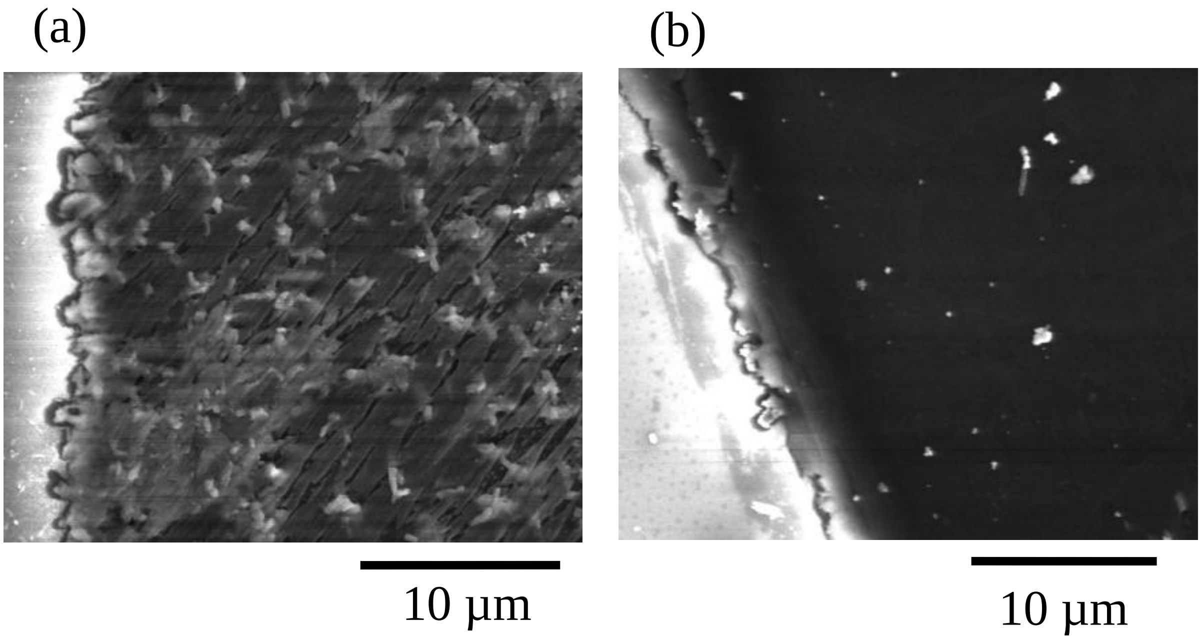

Figure SI8. (a) SEM image of the wire-like morphology area, AgTCNQ section, and (b) SEM image of the smooth area, TCNQ section.
